# Finite Element- and Design of Experiment-Derived Optimization of Screw Configurations and a Locking Plate for Internal Fixation System

**DOI:** 10.1155/2019/5636528

**Published:** 2019-08-21

**Authors:** Wei Sheng, Aimin Ji, Runxin Fang, Gang He, Changsheng Chen

**Affiliations:** ^1^College of Mechanical and Electrical Engineering, Hohai University, Changzhou 213022, China; ^2^Changzhou Orthmed Medical Instrument Co., Ltd., Changzhou 213022, China

## Abstract

**Objectives:**

The optimization for the screw configurations and bone plate parameters was studied to improve the biomechanical performances such as reliable internal fixation and beneficial callus growth for the clinical treatment of femoral shaft fracture.

**Methods:**

The finite element analysis (FEA) of internal fixation system under different screw configurations based on the orthogonal design was performed and so was for the different structural parameters of the locking plate based on the combination of uniform and orthogonal design. Moreover, orthogonal experiment weight matrixes for four evaluation indexes with FEA were analyzed.

**Results:**

The analytical results showed the optimal scheme of screw configuration was that screws are omitted in the thread holes near the fracture site, and single cortical screws are used in the following holes to the distal end, while the double cortical screws are fixed in thread holes that are distal to the fracture; in the other words, the length of the screws showed an increasing trend from the fracture site to the distal end in the optimized configuration. The plate structure was optimized when thread holes gap reached 13 mm, with a width of 11 mm and 4.6 mm and 5 mm for thickness and diameter of the screw, respectively. The biomechanical performance of the internal fixation construct was further improved by about 10% based on the optimal strain range and lower stress in the internal fixation system.

**Conclusions:**

The proposed orthogonal design and uniform design can be used in a more efficient way for the optimization of internal fixation system, which can reduce the simulation runs to about 10% compared with comprehensive test, and the methodology can be also used for other types of fractures to achieve better internal fixation stability and optimal healing efficiency, which may provide a method for an orthopedist in choosing the screw configurations and parameters for internal fixation system in a more efficient way.

## 1. Introduction

The femur bears most load among limb bones in human body which is made up of three major parts named the proximal femur, distal femur, and femoral shaft. The femoral shaft fracture is one of the most common fractures in clinical practice [1, 2]. Transversal and comminuted fractures in femoral shaft are usually caused by high kinetic energy impact, extrusion, etc. In clinical application, the locking plate is commonly used as a fixation of those fractures. The internal fixation of locking plate can reduce the destruction of local blood supply under the requirement of strong fixation, but it was reported that the failure rate of screw or plate was quite high, for about 18% of all the treatment with plate fixation [3–5]. The main reasons for the failure occurring in the plate fixation may be from the improper selection of internal fixation to violation rules of internal fixation, wrong functional exercise, etc. [4, 6].

To figure out the frequent failures in screws and bone plates, several related studies were conducted by researchers around the world. Field et al. [7] found that the selectively reducing number of fixed screws had few impacts on the structural stiffness and bone surface stress of internal fixation system; however, optimized scheme of screw configurations was not obtained. Cui et al [8] reported that the axial strength of the device was not affected by the proximal screw, but in a certain working length of plate, proximal screws determined the strength of the internal fixation system. In Katthagen et al.'s study [9], different plate materials and screws can change the stiffness and limit stress of the locking plate; however, the structural parameters of the plate were not analyzed. Sanders et al [10] found that the length of the plate was more important than the number of screws in terms of structure bending strength.

In the recent years, the finite element method (FEM) has been widely adopted in the study to investigate the failure reasons of implants. Zhang et al. [11] used FEM to explore the distributions of different threaded holes on the locking plate, but different configurations of the screws were not studied. Nourisa et al. [12] used FEM to investigate the influence of screws number and position on the interfragmentary strain of LCP-femur system for a midshaft fracture. Heyland et al. [13] found that semirigid screws could change plate working length to control interfragmentary movement for locking plate fixation at the distal femur. Kim et al. [14] used the Taguchi method and FEM to optimize the geometry of plate and screws; the results showed that the bone screw length was decreased by 60% and the bone plate volume reduced by about 53%, which indicates that the Taguchi method combined with FEM is a useful way for optimization. Okumura et al [15] adopted FEM to explore the influence of cortical layer thickness of bone and structural parameters of the plate on the biomechanical properties of the plate. Chen et al. [16] differentiated the effects of intramedullary nail and locking plate in the treatment of distal femoral fractures with the aid of FEM; however, in his study, the specific parameters of internal fixation system were neglected.

As shown above, the screw configurations and structural parameters of the plate are the two main aspects for study of the fracture treatment. In the multifactor and multilevel study of design of experiments (DOE), the single factor alternative method can be used to only get the best level of the factor in the local area. The result with the full-scale experiment method is relatively reliable, but the cost is too high. The orthogonal experimental design is to select the representative experimental points for experiments, and the full-scale experiment situation can be understood by analyzing results of orthogonal experiment. By using this method, the experimental numbers were greatly reduced, and the best scheme for multiple factors could be obtained [17].

Based on the 3D modeling technique and FEM, DOE (the uniform and orthogonal design of experiments) is used to design the experiments to study the impact of screw configurations and the structural parameters of the locking plate on the biomechanical performance of the internal fixation construct. The weight matrix analysis is applied to estimate the weights of different levels of each factor which affect the stability of internal fixation. The best scheme of screw configurations and optimal parameters of the locking plate are obtained from the methods mentioned above, and the obtained scheme might provide guidance for the orthopedic surgeon while conducting the clinical treatment of femoral shaft fracture fixation.

## 2. Materials and Methods

### 2.1. Geometric Modeling

A pathologically healthy femur from a healthy male (with the informed consent) was scanned by CT (computed tomography). The scanned data were saved in the format of DICOM (Digital Imaging and Communications in Medicine). Then, Mimics 17.0 (Materialise, Leuven, Belgium) was used for data processing to create the surface model of the femur and export in the *∗*.igs format. Prior to the establishment of three-dimensional (3D) model, reverse engineering (RE) software Geomagic Studio 12.0 (Geomagic, North Carolina, USA) was utilized for point cloud processing, surface modification, and 3D model constructing. The structural parameters of the locking plate and the screw in the analysis model were provided by Orthmed Medical Instrument Company (Changzhou, China), and the bone screw and bone plate were substituted by cylinder and plate model for simplification.

### 2.2. Orthogonal Design for Screw Configurations

**Figure 1:**
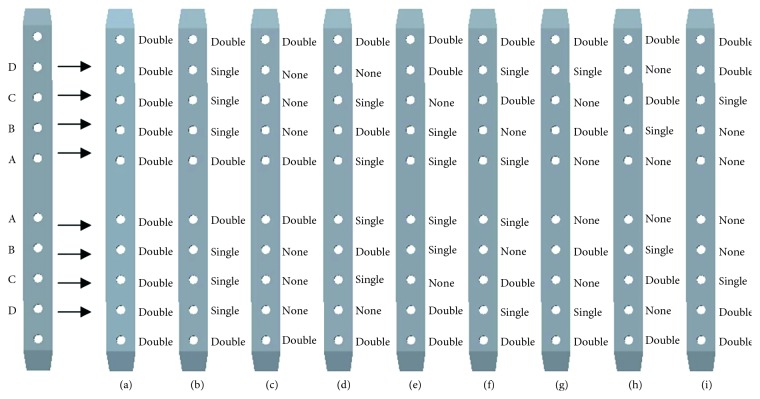
Schematic diagram of four factors and different screw configurations: (a–i) the first type to the ninth type.

The best scheme of the screw configurations can be determined, to ensure the stability of internal fixation. Considering the need of stability for internal fixation system, double cortical screws were used in both sides of distal thread hole to increase the anti-pull-out strength [18]. Therefore, the other four groups of thread holes in the plate were the main focuses in the study. The design factors of four groups of thread holes are shown in Figure 1, where A, B, C, and D represent the screw fixation position in the 4 thread holes from the holes near the fracture to the distal end of fracture site, respectively, and the configurations are set symmetrically to the fracture. As shown in Table 1, each group of fixed screws in the thread holes has 3 levels, respectively. Thus, the L_9_ (3^4^) orthogonal array was used to perform the four factors and three levels of orthogonal experiment in the study of screw configurations, as shown in Table 2 and Figure 1.

In Table 2, calculations of II_1_, III_2_, and Δ_1_ were taken as the example to illustrate the following. II_1_ is the sum of the experimental results of the factor A at the level of “2,” that is, II_1_ = F_4_ + F_5_ + F_6_; III_2_ is the sum of the experimental results for the factor B at the level of “3,” that is, III_2_ = F_3_ + F_6_ + F_9_; Δ_1_ = max{I_1_, II_1_, III_1_} − min{I_1_, II_1_, III_1_}. If the calculation result is Δ_2_ > Δ_3_ > Δ_1_ > Δ_4_, the factor order of influencing on the index is B, C, A, and D [19]. Four indexes of this study were observed as the maximum von Mises stresses of the plate (*P*), screw (*N*), and femur (*K*) and the maximum strain of the callus (*L*). The criteria of better configurations are the one with lower level of the 4 abovementioned indexes.

### 2.3. Finite Element Modeling

The plate, bone, and screw model were assembled in the Pro/E 4.0 (Parametric Technology Corporation, Massachusetts, USA) according to the orthogonal table of L_9_(34), the abovementioned nine types of the internal fixation systems, respectively. Then, the models of the internal fixation were imported into the ANSYS 17.0 (Ansys Inc., Pennsylvania, USA) software; 3 mm thickness fragment was cut out in the middle of the femur to simulate the initial callus state. 10-node tetrahedral element of Solid 187 was used for the model meshed. The cortical bone, cancellous bone, callus, titanium alloy plate, and screws were modeled as continuous, homogeneous, and isotropic linear elastic materials [20], and the corresponding elastic modulus were set as 16.8, 0.84, 0.01, 105, and 105 GPa, respectively, and Poisson's ratio was 0.3 [21–23]. The contacts could be regarded as the glue state as there is no relative movement between the screws and the plate and the screws and the femur [24], same as was the contact state of callus and the femur.

### 2.4. Loading and Solving

A key point above the femoral head should be created to apply conveniently loads acting on the femur. The key point was modeled with 3D Mass21, and the real constant was set. Thus, the master node was established. Then, a rigid area between the master and slave nodes on the surface of the femoral head was further established.

**Figure 2:**
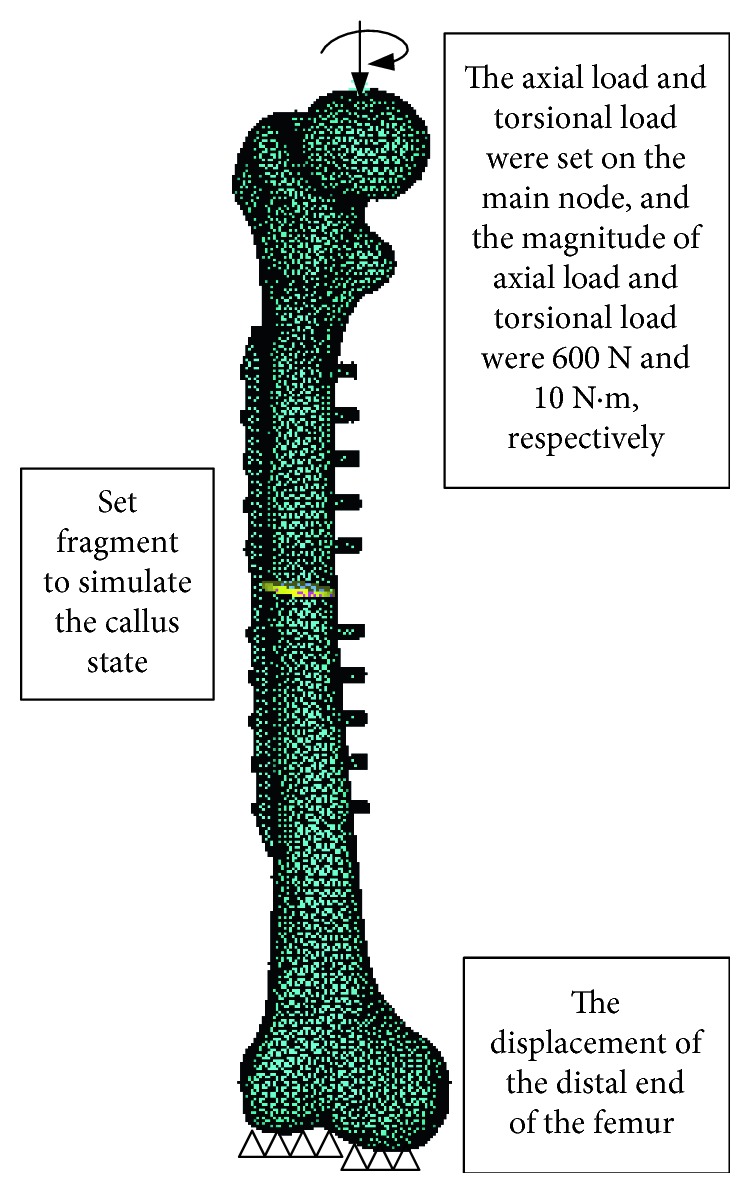
Finite element model of the internal fixation system.

Considering an adult's one leg standing state, the axial compression of 600 N and the torque of 10 N·m were applied to the master node. The distal end of the femur was rigidly fixed, as shown in Figure 2. The corresponding distributions of equivalent stress and strain were obtained after the analysis.

### 2.5. Experimental Design of Structural Parameters of the Plate

To optimize the plate structure, it was necessary to choose the appropriate parameters of plate as design factors and more levels for each factor in DOE. The orthogonal design can be used to search the optimal parameter of plate. And the experimental results were easily performed with intuitionistic analysis or variance analysis. However, the orthogonal design needs relatively more number of experimental runs, at least equal to square of the number of levels. For example, if an experiment has four factors with each factor needing eight levels (the following plate parameters of DOE), one needs at least 8^2^ = 64 experiment runs, which are not included considering the interaction effects between the factors. Therefore, the orthogonal design is not suitable for multilevel case. The uniform design method was proposed to solve the problem. The experimental number of the uniform design could be equal to the number of levels. However, few experimental numbers lead to the result data that need to be processed by regression analysis with the statistical software, e.g., SAS (SAS Institute Inc., North Carolina, USA). Moreover, it demands the analyst to have not only solid professional knowledge and but also considerably plenty of statistical knowledge. To utilize the advantages of the orthogonal design and the uniform design, the combination of two experimental designs [25] was applied in this study, i.e., little number of experimental runs was adopted to obtain the optimal parameters by intuitionistic analysis.

#### 2.5.1. Preliminary Screening of Levels at Each Factor

**Figure 3:**
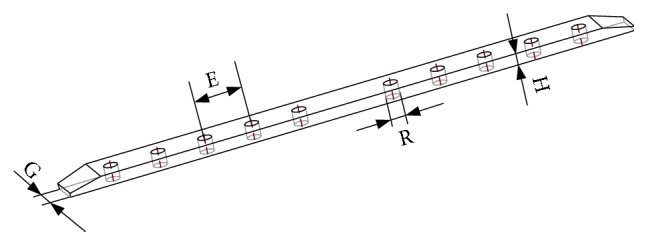
Design factors of the plate.

As for the uniform design, experimental points are uniformly scattered in the domain. The optimal designs in the experiment are close to those over all experimental domain. Thus, the experimental results are effective and accepted. So, the uniform design can be used firstly for preliminary screening of levels for each factor in multiple number of levels and the corresponding orthogonal design for finely screening of optimal experimental parameters. In order to screen their levels, four design factors were selected, and uniform design table of U^*∗*^_8_(8^5^) (Table 3) was used to arrange 8 experiment runs. In Table 3, the four factors of *E*, *G*, *H*, and *R* denote the spacing between the thread holes, the width of the lower surface of the plate, the thickness of the plate, and the diameter of the screw, respectively, as shown in Figure 3. Eight levels were set for each factor, and the maximum level was consistent with the original plate parameter. Then, according to Table 3, each group's finite element model was created, loaded, and solved.

#### 2.5.2. Orthogonal Design of Structural Parameters of the Plate

Because the optimal structural parameters of the plate in the experiment were close to the preliminarily screened experimental results by the uniform design, three levels most close to the experimental levels screened by the uniform design were selected and finely screened for the optimal structural parameters of the plate by the orthogonal design with the L_9_(3^4^) orthogonal array (Table 4).

### 2.6. Experimental Tests

**Figure 4:**
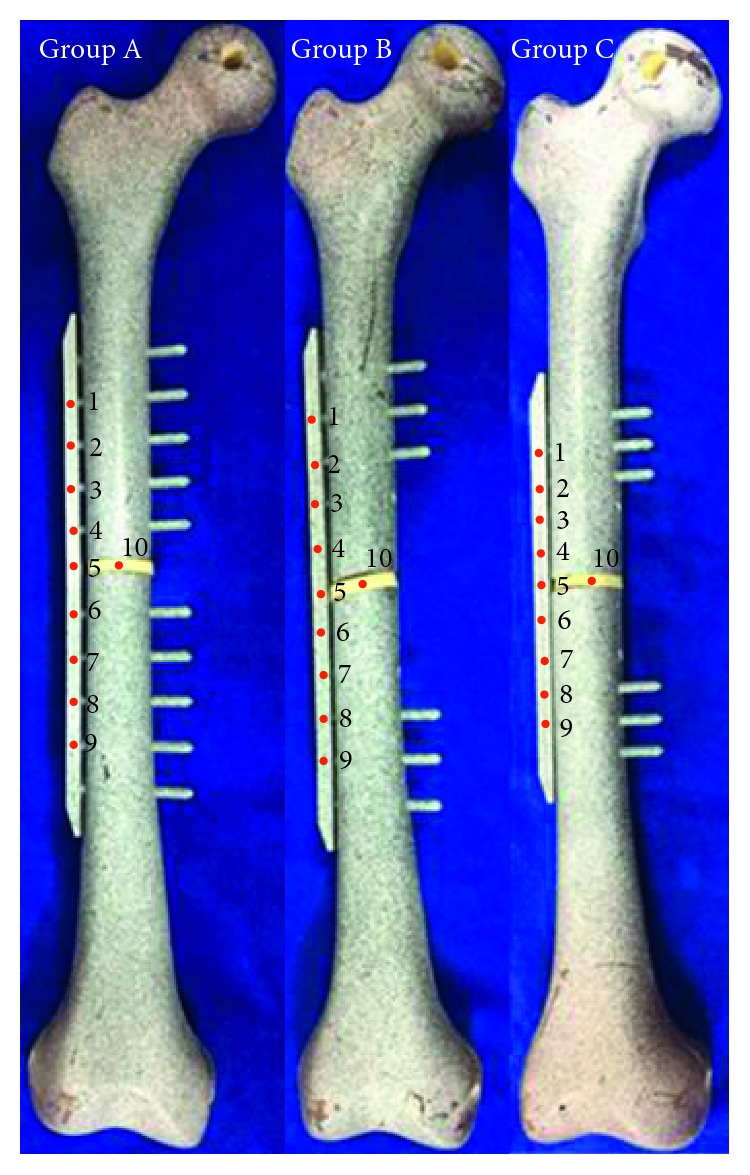
Layout of measuring points on each model.

To check the above experimental design results, physical models for testing were made, in which an artificial bone (Sawbones, Washington, USA) was utilized, as shown in Figures 3 and 4. Three models of different fracture fixation constructs were considered: the original internal fixation model (group A), the internal fixation model with screw configurations optimization (group B), and the internal fixation model with screw configurations and plate optimization (group C). The layout of ten measuring points on each model is shown in Figure 4.

**Figure 5:**
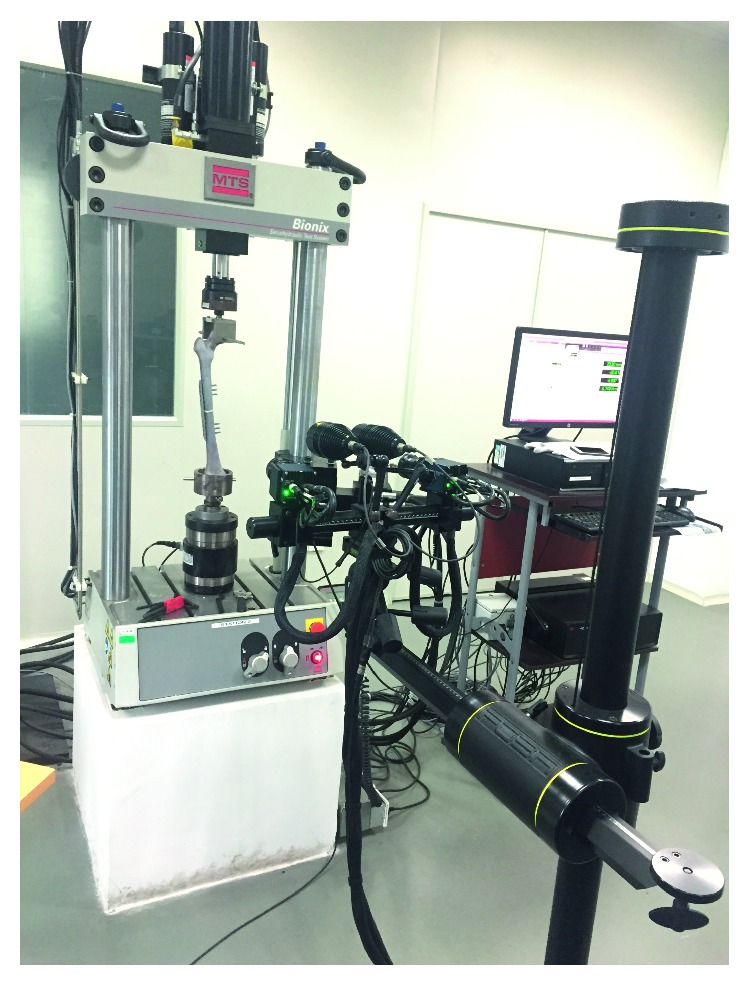
Setup of the internal fixation model.

In Figure 5, the MTS Bionix servohydraulic test system (INSTRON, Massachusetts, USA) was used to perform combined loading. Axial compression loading of 600 N and torsional loading of 10 N·m were applied to the proximal end of the bone. ARAMIS optical system (GOM, Braunschweig, Germany) was used to measure the displacement and velocity under loadings and obtain the strain of each measuring point.

## 3. Results

### 3.1. Screw Configurations

**Figure 6:**
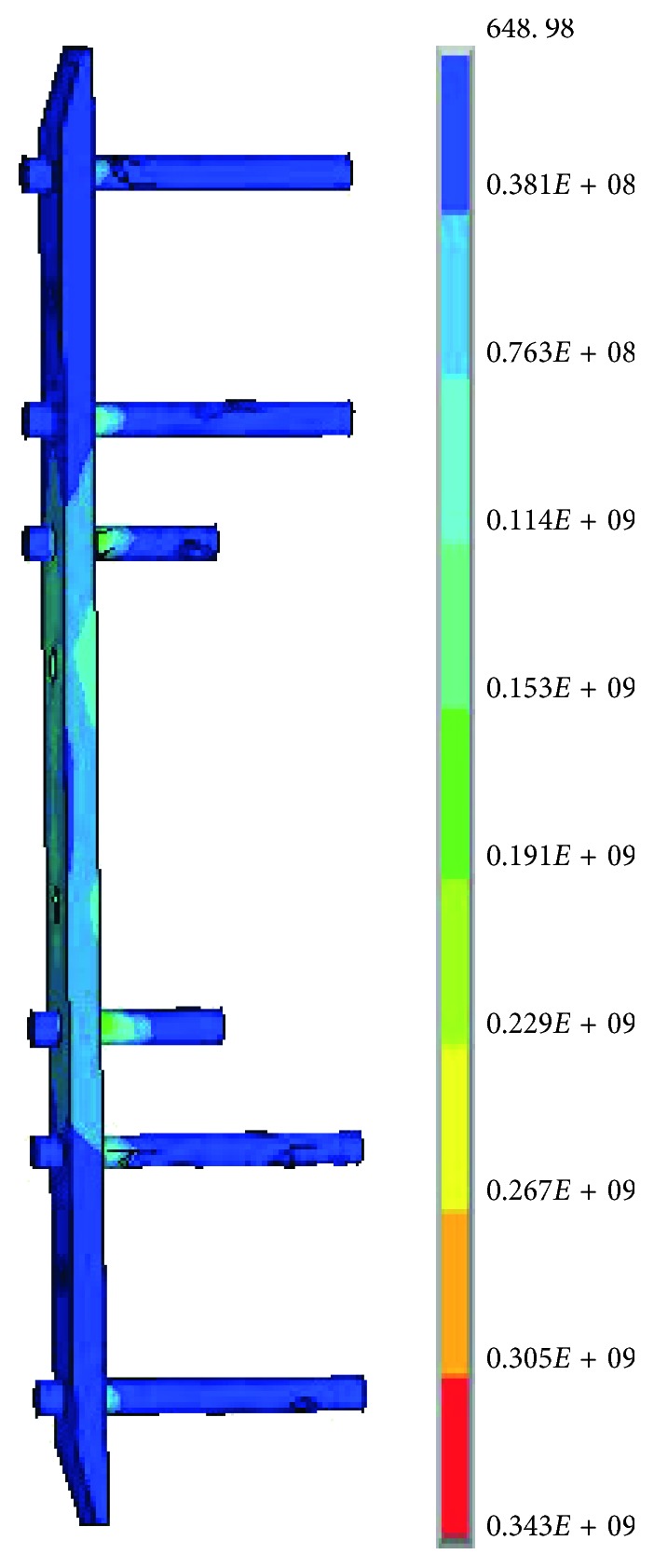
von Mises stress of the implants in the no. 8 group.

**Figure 7:**
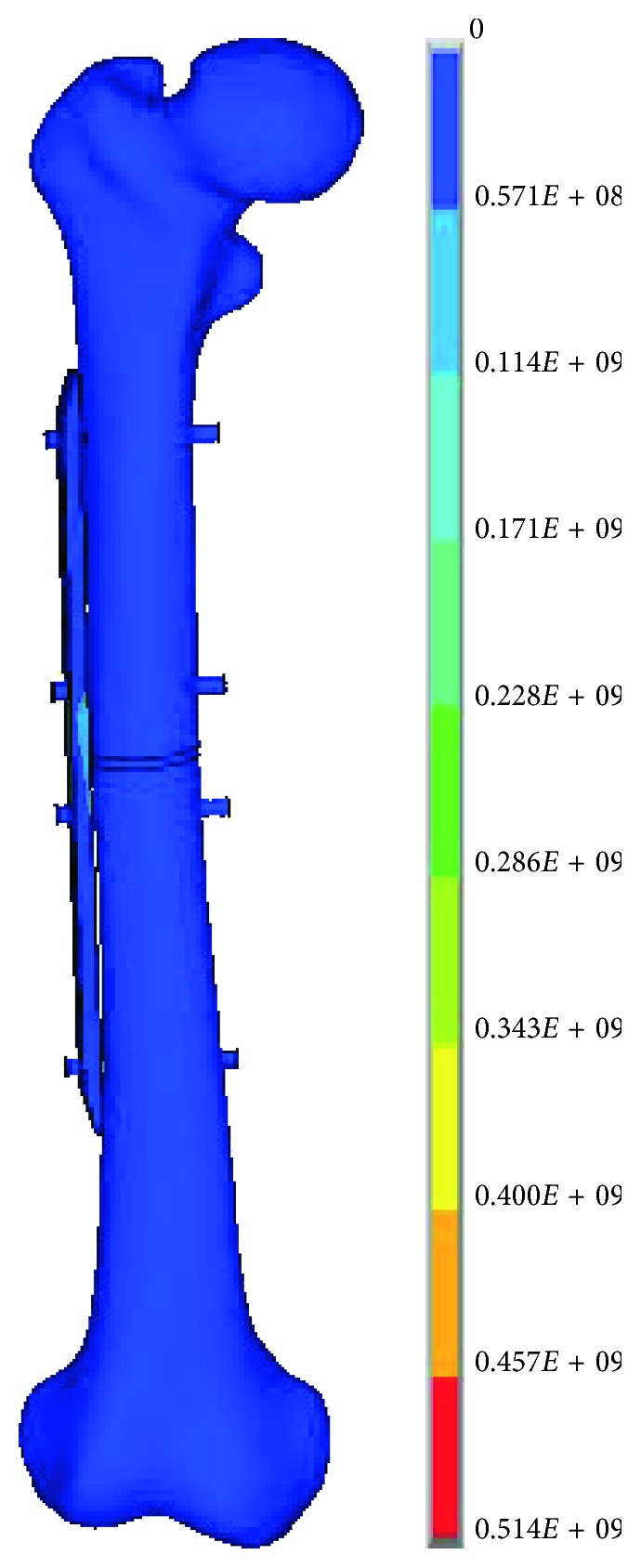
von Mises stress contour of the internal fixation system in the no. 3 group.

As shown in Table 5, the simulation results of four indexes were obtained by the FEA of the nine types of experimental scheme from the orthogonal designs of screw configurations. The maximum von Mises stress of screws at each type was greater than that of the plate. The von Mises stress of the implants at the eighth type was the smallest, as shown in Figure 6. The maximum von Mises stress of the femur at the third type was slightly smaller than that of other types. The maximum strain of callus at each type was close. Among all types, the maximum von Mises stress occurred at the screw in the third type; the von Mises stress contour of its internal fixation model is shown in Figure 7. An intuitionistic analysis was made for the four indexes. The obtained results are shown in Table 6. In this paper, *P*, *N*, and *K* denote the maximum von Mises stress of plate, screw, and the femur, respectively, and *L* denotes the maximum strain in the callus.

From Table 6, the optimal schemes of screw configurations were A_3_B_2_C_2_D_1_, A_3_B_2_C_2_D_1_, A_1_B_2_C_1_D_2_, and A_1_B_2_C_1_D_1_ according to the maximum von Mises stress of the plate (*P*), the screw (*N*), and the femur (*K*) and the maximum strain of callus (*L*), respectively. Due to the different schemes obtained with respect to different index, the comprehensive consideration for the four indexes was needed to achieve the best scheme. In this study, the weight analysis method [26] was used to compute the weight of every level at each factor influencing the indexes, and the best scheme can be determined for the orthogonal experiment according to evaluated weight value. The calculation processes were as follows.

Three layer matrixes and the weight matrix were given by

(1) $$\begin{array}{c} \text{index layer, }M={\left[\begin{array}{cccccccccccc} \frac{1}{{\text{I}}_{1}} & \frac{1}{{\text{II}}_{1}} & \frac{1}{{\text{III}}_{1}} & 0 & 0 & 0 & 0 & 0 & 0 & 0 & 0 & 0\\ 0 & 0 & 0 & \frac{1}{{\text{I}}_{2}} & \frac{1}{{\text{II}}_{2}} & \frac{1}{{\text{III}}_{2}} & 0 & 0 & 0 & 0 & 0 & 0\\ 0 & 0 & 0 & 0 & 0 & 0 & \frac{1}{{\text{I}}_{3}} & \frac{1}{{\text{II}}_{3}} & \frac{1}{{\text{III}}_{3}} & 0 & 0 & 0\\ 0 & 0 & 0 & 0 & 0 & 0 & 0 & 0 & 0 & \frac{1}{{\text{I}}_{4}} & \frac{1}{{\text{II}}_{4}} & \frac{1}{{\text{III}}_{4}} \end{array}\right]}^{T},\\ \text{factor layer, }T=\left[\begin{array}{cccc} \frac{1}{\left(1/{\text{I}}_{1}\right)+\left(1/{\text{II}}_{1}\right)+\left(1/{\text{III}}_{1}\right)} & 0 & 0 & 0\\ 0 & \frac{1}{\left(1/{\text{I}}_{2}\right)+\left(1/{\text{II}}_{2}\right)+\left(1/{\text{III}}_{2}\right)} & 0 & 0\\ 0 & 0 & \frac{1}{\left(1/{\text{I}}_{3}\right)+\left(1/{\text{II}}_{3}\right)+\left(1/{\text{III}}_{3}\right)} & 0\\ 0 & 0 & 0 & \frac{1}{\left(1/{\text{I}}_{4}\right)+\left(1/{\text{II}}_{4}\right)+\left(1/{\text{III}}_{4}\right)} \end{array}\right],\\ \text{lever layer, }S={\left[\begin{array}{cccc} \frac{\Delta_{1}}{\Delta_{1}+\Delta_{2}+\Delta_{3}+\Delta_{4}} & \frac{\Delta_{2}}{\Delta_{1}+\Delta_{2}+\Delta_{3}+\Delta_{4}} & \frac{\Delta_{3}}{\Delta_{1}+\Delta_{2}+\Delta_{3}+\Delta_{4}} & \frac{\Delta_{4}}{\Delta_{1}+\Delta_{2}+\Delta_{3}+\Delta_{4}} \end{array}\right]}^{T}. \end{array}$$

The weight matrix *ω*=*M* · *T* · *S*.

There were four respective weights obtained for the four indexes in Table 6. The four weights were averaged as

(2) $$\begin{array}{c} \omega =\frac{\omega_{1}+\omega_{2}+\omega_{3}+\omega_{4}}{4}\\ =\frac{1}{4}\left(\left[\begin{array}{c} 0.16962\\ 0.17142\\ 0.23999\\ 0.024130\\ 0.025153\\ 0.024791\\ 0.085923\\ 0.10055\\ \begin{array}{c} 0.086671\\ 0.024496\\ 0.023738\\ 0.023525 \end{array} \end{array}\right]+\left[\begin{array}{c} \begin{array}{c} 0.087918 \end{array}\\ 0.097330\\ 0.10247\\ 0.13378\\ 0.14336\\ 0.11633\\ 0.048422\\ 0.049130\\ \begin{array}{c} 0.045528\\ 0.062222\\ 0.056965\\ 0.056552 \end{array} \end{array}\right]+\left[\begin{array}{c} 0.11776\\ 0.10235\\ 0.11571\\ 0.055246\\ 0.059430\\ 0.056965\\ 0.062186\\ 0.058234\\ \begin{array}{c} 0.057619\\ 0.10350\\ 0.11258\\ 0.098426 \end{array} \end{array}\right]+\left[\begin{array}{c} 0.033285\\ 0.033630\\ 0.032479\\ 0.10164\\ 0.11385\\ 0.10694\\ 0.14538\\ 0.12740\\ \begin{array}{c} 0.12601\\ 0.062113\\ 0.058983\\ 0.058297 \end{array} \end{array}\right]\right)=\left[\begin{array}{c} 0.12390\\ 0.11850\\ 0.13860\\ 0.07195\\ 0.07573\\ 0.07162\\ 0.07530\\ 0.07455\\ \begin{array}{c} 0.07525\\ 0.05914\\ 0.05903\\ 0.05638 \end{array} \end{array}\right]=\left[\begin{array}{c} \omega_{A_{1}}\\ \omega_{A_{2}}\\ \omega_{A_{3}}\\ \omega_{B_{1}}\\ \omega_{B_{2}}\\ \omega_{B_{3}}\\ \omega_{C_{1}}\\ \omega_{C_{2}}\\ \begin{array}{c} \omega_{C_{3}}\\ \omega_{D_{1}}\\ \omega_{D_{2}}\\ \omega_{D_{3}} \end{array} \end{array}\right]. \end{array}$$

As shown in the above expression, the weights of three levels at factor A were 0.1239, 0.1185, and 0.1386, respectively, where the weight of A_3_ is the largest in factor A. Similarly, the weights of B_2_, C_1_, and D_1_ are the largest in factor B, C, and D, respectively. Because the weights of A_3_, B_2_, C_1_, and D_1_ are the largest corresponding to factors A, B, C, and D, the best scheme in the orthogonal experiment is A_3_B_2_C_1_D_1_, which implied that the screws were omitted in A holes, single cortical screws were fixed in B thread holes, and double cortical screws were fixed in C and D thread holes, that is to say, optimal screw configurations are the type with screws that are omitted in the thread holes near the fracture site, and single cortical screws are fixed in the following holes to the distal end, while the double cortical screws are used in thread holes that are distal to the fracture. Meanwhile, the influences of the factors on the indexes were A, C, B, and D in order based on the weight value of the four factors.

### 3.2. Preliminary Screening of Structural Parameters of the Plate

**Figure 8:**
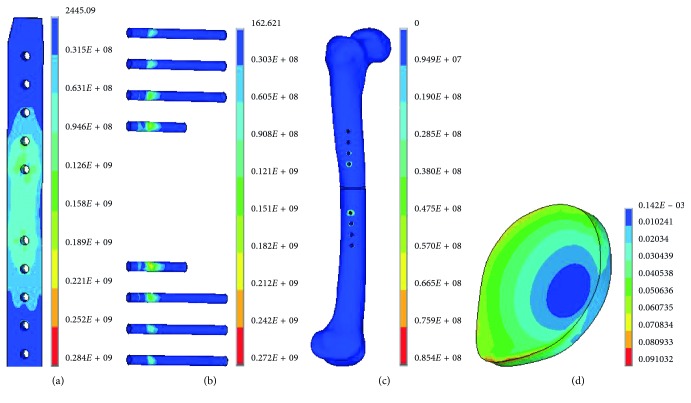
The von Mises stress contour of the plate (a), the screw, (b) and the femur (c) and the strain contour of the callus (d) in the no. 4 group.

After finishing FEA of the eight types from the uniform design of structure parameters, the simulation results of four indexes were obtained, as shown in Table 7. Considering the optimal fracture healing range was 2%–10% and the maximum von Mises stress for the femur should not exceed 100 MPa, the fourth type of uniform experiments was screened as the optimal scheme. As shown in Table 3, in row 4, the optimal plate parameters were that the spacing between the thread hole is 14 mm, the width of the lower surface of the plate is 12 mm, the thickness of the plate is 4.9 mm, and the diameter of screw is 4.5 mm. The von Mises stress contour of the plate, the screw, and the femur and the strain contour of the callus in the fourth type of experiments are shown in Figure 8.

### 3.3. Orthogonal Design of Structural Parameters of the Plate

**Figure 9:**
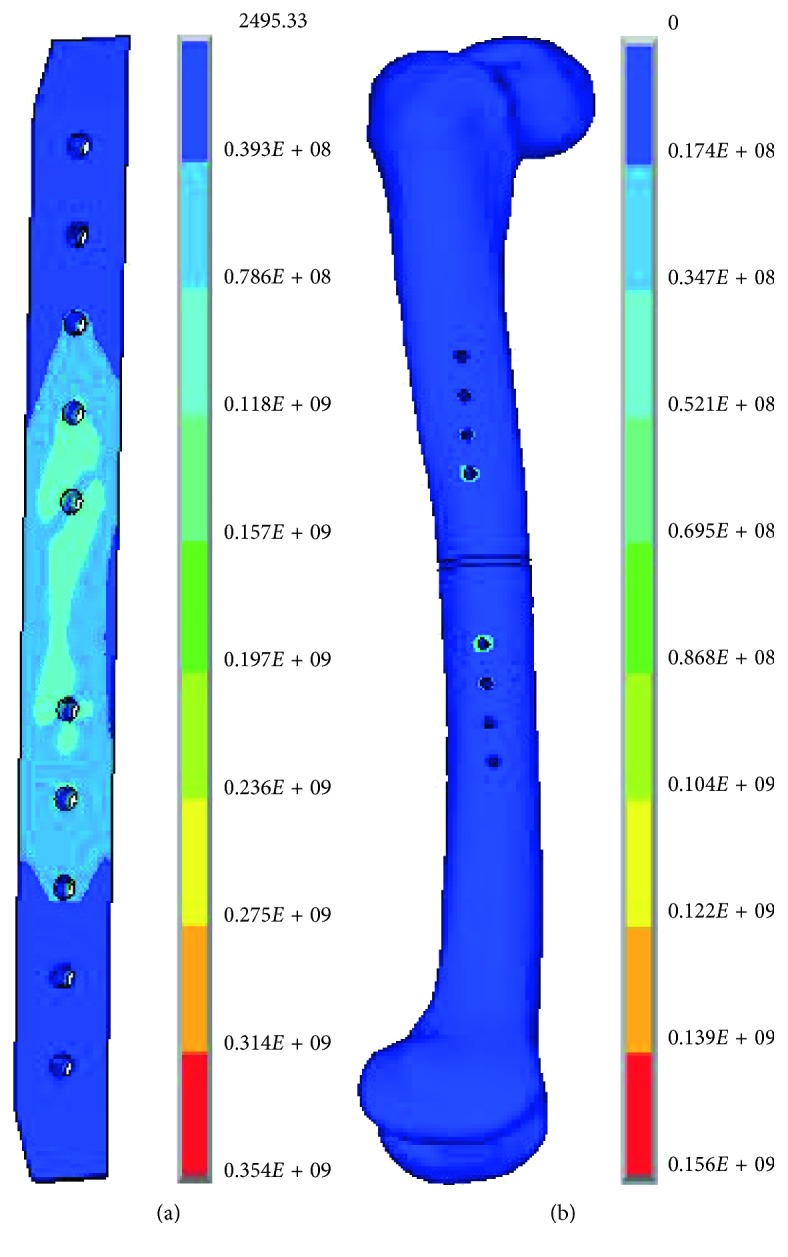
The von Mises stress contour of the plate (a) and the femur (b) in the no. 9 group.

Three levels close to the optimal plate parameters by the above uniform design were determined: let *E* be 13, 14, and 15 mm, *G* be 11, 11.5, and 12 mm, *H* be 4.6, 4.9, and 5.2 mm, and *R* be 4, 4.5, and 5 mm. The orthogonal design with the L_9_(3^4^) array (Table 4) was carried out. The simulation results of four indexes were obtained by the FEA of the nine types of experimental scheme from the orthogonal design of structure parameters (Table 8). The largest value for the maximum von Mises stress of the implant and the femur occurred in the ninth type. And the maximum von Mises stress of the femur reached 156 MPa, much larger than that of the other type. The maximum strain of callus at each type was close. The von Mises stress contours of the plate and the femur in the ninth type are shown in Figure 9. An intuitionistic analysis was made for the four indexes. The obtained results are listed in Table 9.

For the abovementioned screw configurations, weight matrix analysis was applied to obtain the weights for three levels at four factors. According to Table 9, the results were given by

(3) $$\begin{array}{c} \omega =\frac{\omega_{1}+\omega_{2}+\omega_{3}+\omega_{4}}{4}\\ =\frac{1}{4}\left(\left[\begin{array}{c} 0.061551\\ 0.056137\\ 0.056705\\ 0.060848\\ 0.060202\\ 0.055551\\ 0.035035\\ 0.035282\\ \begin{array}{c} 0.033436\\ 0.15435\\ 0.18652\\ 0.20439 \end{array} \end{array}\right]+\left[\begin{array}{c} \begin{array}{c} \text{0.}057580 \end{array}\\ \text{0.}054366\\ \text{0.}056513\\ \text{0.}060559\\ \text{0.}058054\\ \text{0.}057014\\ 0.031469\\ 0.030485\\ \begin{array}{c} 0.031236\\ 0.16914\\ 0.18866\\ 0.20493 \end{array} \end{array}\right]+\left[\begin{array}{c} 0.088351\\ 0.087279\\ 0.068494\\ 0.033922\\ 0.035481\\ 0.031819\\ 0.076226\\ 0.061037\\ \begin{array}{c} 0.065495\\ 0.11536\\ 0.14783\\ 0.18870 \end{array} \end{array}\right]+\left[\begin{array}{c} 0.069412\\ 0.067450\\ 0.064706\\ 0.022983\\ 0.22884\\ 0.22443\\ 0.10107\\ 0.10390\\ \begin{array}{c} 0.11306\\ 0.12602\\ 0.14082\\ 0.14526 \end{array} \end{array}\right]\right)=\left[\begin{array}{c} 0.069224\\ 0.066308\\ 0.061605\\ 0.044578\\ 0.044155\\ 0.041707\\ 0.060950\\ 0.057676\\ \begin{array}{c} 0.060806\\ 0.14122\\ 0.16596\\ 0.18582 \end{array} \end{array}\right]=\left[\begin{array}{c} \omega_{E_{1}}\\ \omega_{E_{2}}\\ \omega_{E_{3}}\\ \omega_{G_{1}}\\ \omega_{G_{2}}\\ \omega_{G_{3}}\\ \omega_{H_{1}}\\ \omega_{H_{2}}\\ \begin{array}{c} \omega_{H_{3}}\\ \omega_{R_{1}}\\ \omega_{R_{2}}\\ \omega_{R_{3}} \end{array} \end{array}\right] \end{array}$$

The best scheme in the orthogonal experiment was E_1_G_1_H_1_R_3_ based on the weights of the factor in the above expression. The optimal parameters of the plate were that the spacing between the thread holes, the width of the lower surface of the plate, the thickness of the plate, and the diameter of the screw were 13, 11, 4.6, and 5 mm, respectively. The influences of the factors on the indexes were R, E, H, and G in order.

### 3.4. Optimization Evaluation

Biomechanical performance of optimal scheme that we obtained based on DOE was compared with the original construct, and the stress in the plate and screws was used to evaluate the improvement; the comparison is shown in Table 10. 

The maximum stress in the femur was all lower than 100 MPa in the original and optimal constructs, and the maximum strain in the callus was both in the optimal range of 2%–10% for the two schemes. And the maximum stress in the plate and screws was reduced by 10.9% and 12.7%, respectively, after the optimization. The conclusion could be made that the stability of internal fixation system was improved by about 10% using the optimization.

### 3.5. Experimental Validation

After finishing the experimental testing of the above three groups of internal fixation models, the strain at each measuring point of the model was obtained.

**Figure 10:**
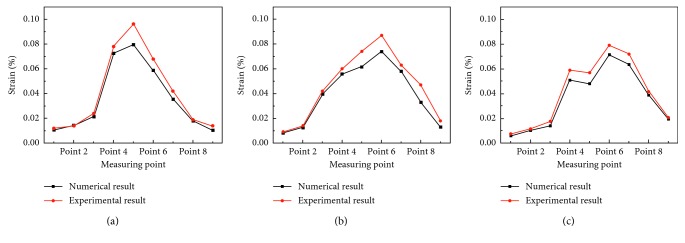
Comparison of numerical predictions of the strain with physical experimental results in (a) Group A, (b) Group B, and (c) Group C.

Figure 10 shows the comparison of the numerical predictions of the strain with the physical experimental results at nine measuring points. The maximum difference between simulation results and experimental results was 17.083%, which occurred at Point 5 in Group A.

The measuring point 10 was located on the simulated callus in Groups A–C. The strains were 5.342%, 4.653%, and 3.501%, respectively. The strain from corresponding FEA results was 5.422%, 4.662%, and 3.280%. The maximum deviation rate of the all combinations was 6.312%, which occurred in Group C.

## 4. Discussion

In this study, the optimal design of internal fixation parameters was studied to achieve the stability and healing efficiency for the plate fixation system. Screw configurations were optimized by orthogonal design, and the plate dimensions were optimized by the combination of uniform and orthogonal design. Parameter combinations for each simulation were determined by uniform and orthogonal table. The criteria used for evaluating the optimal construct were fixation stability and healing performance for the fractured bone.

For the optimization of screw configurations using intuitionistic analysis for orthogonal design, the stress in the internal fixation construct and femur was greatly influenced by the first and second holes that are near the fracture site. The result is in accordance with previous studies [27, 28], which indicates that the usage of screw hole near the fracture site has great influence on the stress distribution in the internal fixation system. However, when it comes to the index of callus strain, the third screw hole that is far from the fracture site had the biggest influence, which is contradictory to some previous studies [29, 30], and may result from our focus on the length of screws and not the other variables such as working length, screw density, and so on. In a word, different schemes were obtained under different evaluation indexes, and these schemes may be contradicted. The weight matrix analysis was performed to obtain a comprehensive consideration for these different evaluation indexes. The optimal construct of screw length in different holes showed that screws should be omitted in the first thread holes near the fracture site; single cortical screws are used in the following holes to the distal end, while the double cortical screws are fixed in thread holes that are distal to the fracture. In the other words, the length of the screws showed an increasing trend from the fracture site to the distal end in the optimized configurations. The positive gradient of screw length from fracture site to the distal end is an optimal configuration that can both ensure the fixation stability and healing efficiency. This conclusion is useful for an orthopedist to choose the length of screws and their locations in the plate, and a combination of short screw near the fracture site and long screw length in the distal end can get a balance between the fixation stability and healing efficiency.

For the optimization of plate structure, using orthogonal design may directly not get the ideal combinations, for there exists large number of combinations in the continuous range for structure parameters; a small step range was needed to get the ideal combinations, e.g., each five design parameters has eight levels, which will generate 8^5^ mathematical combinations. The proposed method of design of experiment will greatly reduce the number of runs to less than 10% of comprehensive test. A standard uniform design using the uniform table of U^*∗*^ 8(8^5^) was performed to conduct 8 numerical simulations. Uniform design was used as the preliminary screening for structure parameters of plate system, and the screened parameters were used as the base data for the following orthogonal design. The following orthogonal design showed that screw diameter was the most significant parameter influencing the maximum stress in plate fixation system and strain in the callus. Previous studies [31, 32] have also declared that screw diameter played an important role in the mechanical environment for internal fixation system. Weight matrix analysis showed that the optimal combination was 13 mm, 11 mm, 4.6 mm, and 5 mm for thread hole gap, width of lower surface of the plate, plate thickness, and screw diameters, respectively. The evaluation indexes for these combinations were also beneficial for fixation stability and healing efficiency.

The number by the orthogonal design of 4 levels and 3 factors was reduced to 9 experiment runs, and every level of each factor was combined with every level of the other factors only once in the experiment [28] and so was the uniform design, which can greatly reduce the number of simulations. The influences of the factors on stability of the internal fixation in order and the interactions among the factors can be observed with the intuitionistic analysis. Moreover, the effect weight of every level for each factor on the comprehensive performance index of the internal fixation, the best scheme, and the influences of the factors on the experimental results in order can be obtained by combining the weight matrix analysis with the intuitionistic analysis in this study.

Although the orthogonal design can be used to arrange the experiments of multiple factors and significantly reduce the number of experiments, more levels of factors usually appeared in the experiment of biomedical engineering, which leads to much more experiment runs. Uniform design is suitable for multifactor and multilevel DOE and can further reduce number of experiments. In order to improve larger number of experiments for orthogonal design and the result data being difficult to handle for uniform design, two methods were combined in this study. In the parameter optimization of plate, at least 8^5^ experimental runs were reduced to 17 runs by utilizing the combined experiment method. Thus, the number of experiments was further reduced, which was more convenient for the result data processing. The combined method proposed in the present study is suitable for optimization of medical implant parameters, biological material properties, production formula, etc., which could also be used for other scenarios of fractures to achieve better internal fixation stability and improved healing efficiency.

Moreover, the finite element models were validated based on the comparison between silicon results and physical test. The deviations of the two results are all lower than 18% under the three combinations, which means that the numerical model is acceptable for physical test. However, the comparison results showed that strain from numerical simulation in the measuring points are all higher than that from physical test. This may be resulted from the ideal boundary condition and contact pairs in the silicon research. The ideal boundary conditions may ignore some restrictions like the contact distance between bone and the plate, the different locations of thread screws for physical models, and so on. All these defections would cause larger deformation in the numerical models. At the same time, the deviations are large at the measuring points of 4, 5, 6, and 7. These points are all located among the working length of the plate, where the region is believed to generate large deformations. Due to the ideal boundary conditions for numerical simulations, the deviation would be enlarged under the higher deformation compared with the measuring points that are far from the fracture site.

There was generally consistent trend between the numerical predictions of the strain and the measuring values in Group A, Group B, and Group C, as shown in Figure 10. The deviation of results might be due to difficulty in the FEA model agreement with the physical model. For example, there was the difference between human bone and artificial bone. In addition, loading and boundary condition of FEA models were idealized compared to reality.

Limitations of the study were simplification for the FE modeling of the internal fixation and its specific load applied on the femur. Firstly, the femur was modeled as two uniform homogeneous materials in the present study, whereas the femur material is heterogeneous. Secondly, two simplified loads of compression and torsion were applied to the FE model without considering the effect of muscle, and the magnitude of these loads are limited to a specific type (600 N for compression and 10 N·m for torque), while the other load types can lead to the different results for internal fixation system; this is necessary to mention that the conclusion obtained in this paper corresponds to a patient of 60 kg only in standing position, and the other load types need to be conducted rapidly with the help from the methods mentioned in this paper. In addition, the distal end of the femur was fully constrained in boundary conditions of the model, whereas physiologically there is partial constraint. Additionally, the criterion we used for evaluation bone healing is the interfragmentary theory, which did not consider the whole healing process, but only the initial condition for the bone healing. For better evaluation of the healing performance, a combination of deviation strain and fluid flow is useful stimuli [17, 33]. Blood vessel growth [34] and cell proliferation [35] are also important factors that are needed to be considered in the fractured bone healing. Moreover, different orthopedic implants such as intramedullary nails [35, 36] will provide different mechanical environments for fracture site during bone healing. Thus, our future work would focus on these limitations of the study.

## 5. Conclusions

The present study presents a design of an experimental method to conduct an optimization for internal fixation system with the plate. The efficiency of optimization for screw configurations and plate structure was greatly improved with the large number of runs reduced. The optimized results will provide enhanced biomechanical environment for internal fixation stability and optimal healing efficiency under the specific fracture case, and the other cases could also be optimized efficiently with this methodology. Furthermore, the present approach could provide guidance in the choice of internal fixation parameters in a more efficient way, which could also lead to the exploration of modification and optimization for internal fixation system.

## Figures and Tables

**Table 1:** Factors and levels.

Lever	Factor
	Different screw fixations in A, B, C, or D thread holes
1	Double cortical
2	Single cortical
3	Screw omission

Double cortical screws: threaded in the both side of cortical bones. Single cortical screws: threaded in the single side of cortical bones.

**Table 2:** Orthogonal array of 4 factors and 3 levels and the analysis of experimental results.

Number of experiments	Factors				Results of experiment F
	A	B	C	D	
1	A_1_	B_1_	C_1_	D_1_	F_1_
2	A_1_	B_2_	C_2_	D_2_	F_2_
3	A_1_	B_3_	C_3_	D_3_	F_3_
4	A_2_	B_1_	C_2_	D_3_	F_4_
5	A_2_	B_2_	C_3_	D_1_	F_5_
6	A_2_	B_3_	C_1_	D_2_	F_6_
7	A_3_	B_1_	C_3_	D_2_	F_7_
8	A_3_	B_2_	C_1_	D_3_	F_8_
9	A_3_	B_3_	C_2_	D_1_	F_9_

Analysis of experimental results	I_1_	I_2_	I_3_	I_4_	
	II_1_	II_2_	II_3_	II_4_	
	III_1_	III_2_	III_3_	III_4_	
	Δ_1_	Δ_2_	Δ_3_	Δ_4_	

**Table 3:** Uniform design of structural parameters of the plate.

Number of experiments	Factors				Results of experiment F
	*E* (mm)	*G* (mm)	*H* (mm)	*R* (mm)	
1	11	9	4	5	F_1_
2	12	10	5.2	5	F_2_
3	13	11	3.7	4.5	F_3_
4	14	12	4.9	4.5	F_4_
5	15	8.5	3.4	4	F_5_
6	16	9.5	4.6	4	F_6_
7	17	10.5	3.1	3.5	F_7_
8	18	11.5	4.3	3.5	F_8_

**Table 4:** Orthogonal array of optimal structural parameters.

Number of experiments	Factors				Results of experiment F
	E	G	H	R	
1	E_1_	G_1_	H_1_	R_1_	F_1_
2	E_1_	G_2_	H_2_	R_2_	F_2_
3	E_1_	G_3_	H_3_	R_3_	F_3_
4	E_2_	G_1_	H_2_	R_3_	F_4_
5	E_2_	G_2_	H_3_	R_1_	F_5_
6	E_2_	G_3_	H_1_	R_2_	F_6_
7	E_3_	G_1_	H_3_	R_2_	F_7_
8	E_3_	G_2_	H_1_	R_3_	F_8_
9	E_3_	G_3_	H_2_	R_1_	F_9_

Processing of experimental results	I_1_	I_2_	I_3_	I_4_	
	II_1_	II_2_	II_3_	II_4_	
	III_1_	III_2_	III_3_	III_4_	

**Table 5:** FEA results for the orthogonal experiment of screw configurations.

Number of experiments	*P* (MPa)	*N* (MPa)	*K* (MPa)	*L* (%)
1	299	393	69.6	7.23
2	257	396	63	7.75
3	300	514	76.7	8.41
4	267	383	88.7	8.65
5	283	350	80.3	7.36
6	297	444	71.8	7.14
7	221	391	70.4	8.92
8	215	343	69.3	7.03
9	169	384	73.3	8.02

**Table 6:** Intuitionistic analysis for the orthogonal experiment of screw configurations.

Number of experiments	Factors				Results of experiment F			
	A	B	C	D	P	N	K	L
1	A_1_	B_1_	C_1_	D_1_	P_1_	N_1_	K_1_	L_1_
2	A_1_	B_2_	C_2_	D_2_	P_2_	N_2_	K_2_	L_2_
3	A_1_	B_3_	C_3_	D_3_	P_3_	N_3_	K_3_	L_3_
4	A_2_	B_1_	C_2_	D_3_	P_4_	N_4_	K_4_	L_4_
5	A_2_	B_2_	C_3_	D_1_	P_5_	N_5_	K_5_	L_5_
6	A_2_	B_3_	C_1_	D_2_	P_6_	N_6_	K_6_	L_6_
7	A_3_	B_1_	C_3_	D_2_	P_7_	N_7_	K_7_	L_7_
8	A_3_	B_2_	C_1_	D_3_	P_8_	N_8_	K_8_	L_8_
9	A_3_	B_3_	C_2_	D_1_	P_9_	N_9_	K_9_	L_9_

I	285.33	262.33	270.33	250.33	The maximum von Mises stress of the plate (*P*)			
II	282.33	251.67	231.00	258.33				
III	201.67	255.33	268.00	260.67				
Range	83.67	10.67	39.33	10.33				
Optimal scheme	A_3_	B_2_	C_2_	D_1_				

I	434.33	389.00	393.33	375.67	The maximum von Mises stress of the screw (*N*)			
II	392.33	363.00	387.67	410.33				
III	372.67	447.33	418.33	413.33				
Range	61.67	84.33	30.67	37.67				
Optimal scheme	A_3_	B_2_	C_2_	D_1_				

I	69.77	76.23	70.23	74.40	The maximum von Mises stress of the femur (*K*)			
II	80.27	70.87	75.00	68.40				
III	71.00	73.93	75.80	78.23				
Range	10.50	6.36	5.57	9.83				
Optimal scheme	A_1_	B_2_	C_1_	D_2_				

I	7.71	8.27	7.13	7.54	The maximum strain of the callus (*L*)			
II	7.72	7.38	8.14	7.94				
III	7.99	7.86	8.23	8.03				
Range	0.28	0.89	1.10	0.49				
Optimal scheme	A_1_	B_2_	C_1_	D_1_				

**Table 7:** FEA results for the uniform experiment of structural parameters of the plate.

Number of experiments	*P* (MPa)	*N* (MPa)	*K* (MPa)	*L* (%)
1	265	245	102	11.39
2	268	250	128	8.39
3	269	257	100	12.47
4	284	272	85.4	9.10
5	331	293	148	15.16
6	336	282	208	11.32
7	328	346	174	15.96
8	325	339	164	12.59

**Table 8:** FEA results for the orthogonal experiment of structural parameters of the plate.

Number of experiments	*P* (MPa)	*N* (MPa)	*K* (MPa)	*L* (%)
1	306	280	102	9.30
2	249	269	92.1	8.12
3	264	246	74.6	7.33
4	248	252	76.6	8.07
5	349	309	113	8.63
6	301	281	82.4	8.77
7	285	256	115	7.98
8	250	244	75.6	8.71
9	354	310	156	9.86

**Table 9:** Intuitionistic analysis for the orthogonal experiment of structural parameters of the plate.

Number of experiments	Factor				Results of experiment F			
	E	G	H	R	P	N	K	L
1	E_1_	G_1_	H_1_	R_1_	P_1_	N_1_	K_1_	L_1_
2	E_1_	G_2_	H_2_	R_2_	P_2_	N_2_	K_2_	L_2_
3	E_1_	G_3_	H_3_	R_3_	P_3_	N_3_	K_3_	L_3_
4	E_2_	G_1_	H_2_	R_3_	P_4_	N_4_	K_4_	L_4_
5	E_2_	G_2_	H_3_	R_1_	P_5_	N_5_	K_5_	L_5_
6	E_2_	G_3_	H_1_	R_2_	P_6_	N_6_	K_6_	L_6_
7	E_3_	G_1_	H_3_	R_2_	P_7_	N_7_	K_7_	L_7_
8	E_3_	G_2_	H_1_	R_3_	P_8_	N_8_	K_8_	L_8_
9	E_3_	G_3_	H_2_	R_1_	P_9_	N_9_	K_9_	L_9_

I	273.00	279.67	285.67	336.33	The maximum von Mises stress of the plate			
II	299.33	282.67	283.67	278.33				
III	296.33	306.33	299.33	254.00				
Range	26.33	26.66	15.66	82.33				
Optimal scheme	E_1_	G_1_	H_2_	R_3_				

I	265.00	262.67	268.33	299.67	The maximum von Mises stress of the screw			
II	280.67	274.00	277.00	268.67				
III	270.00	279.00	270.33	247.33				
Range	15.67	16.33	8.67	52.34				
Optimal scheme	E_1_	G_1_	H_1_	R_3_				

I	89.57	97.87	86.67	123.67	The maximum von Mises stress of the femur			
II	90.67	93.57	108.23	96.50				
III	115.53	104.33	100.87	75.60				
Range	25.96	10.76	21.56	48.07				
Optimal scheme	E_1_	G_2_	H_1_	R_3_				

I	8.25	8.45	8.93	9.26	The maximum strain of the callus			
II	8.49	8.49	8.68	8.29				
III	8.85	8.65	7.98	8.04				
Range	0.60	0.20	0.95	1.22				
Optimal scheme	E_1_	G_1_	H_3_	R_3_				

**Table 10:** Comparison of the optimal construct indexes with those of the original construct.

Construct	Indexes			
	*P* (MPa)	*N* (MPa)	*K* (MPa)	*L* (%)
Original	321	228	86.9	7.16
Optimal	286	199	91.3	9.71

## Data Availability

No data were used to support this study.

## References

[1] Fakoor M., Mousavi S., Javherizadeh H. (2011). Different types of femoral shaft fracture; different types of treatment: their effects on postoperative lower limb discrepancy. *Polish Journal of Surgery*.

[2] Emamhadi M., Saberi A., Andalib S. (2016). Sciatic nerve injuries following femoral shaft fractures: does the time interval from injury to surgery matter?. *Clinical Neurology And Neurosurgery*.

[3] Cronier P., Pietu G., Dujardin C., Bigorre N., Ducellier F., Gerard R. (2010). The concept of locking plates. *Orthopaedics & Traumatology: Surgery & Research*.

[4] Nassiri M., Macdonald B., O’Byrne J. M. (2013). Computational modelling of long bone fractures fixed with locking plates—how can the risk of implant failure be reduced?. *Journal of Orthopaedics*.

[5] Nassiri M., MacDonald B., O’Byrne J. M. (2012). Locking compression plate breakage and fracture non-union: a finite element study of three patient-specific cases. *European Journal of Orthopaedic Surgery & Traumatology*.

[6] Tan S. L. E., Balogh Z. J. (2009). Indications and limitations of locked plating. *Injury*.

[7] Field J. R., Törnkvist H., Hearn T. C., Sumner-Smith G., Woodside T. D. (1999). The influence of screw omission on construction stiffness and bone surface strain in the application of bone plates to cadaveric bone. *Injury*.

[8] Cui S., Bledsoe J. G., Israel H., Watson J. T., Cannada L. K. (2014). Locked plating of comminuted distal femur fractures. *Journal of Orthopaedic Trauma*.

[9] Katthagen J. C., Schwarze M., Warnhoff M., Voigt C., Hurschler C., Lill H. (2016). Influence of plate material and screw design on stiffness and ultimate load of locked plating in osteoporotic proximal humeral fractures. *Injury*.

[10] Sanders R., Haidukewych G. J., Milne T., Dennis J., Latta L. L. (2002). Minial versus maximal plate fixation technique of the ulna: the biomechanical effect of number of screws and plate length. *Journal of Orthopaedic Trauma*.

[11] Zhang Y.-K., Wei H.-W., Lin K.-P., Chen W.-C., Tsai C.-L., Lin K.-J. (2016). Biomechanical effect of the configuration of screw hole style on locking plate fixation in proximal humerus fracture with a simulated gap: a finite element analysis. *Injury*.

[12] Nourisa J., Baseri A., Sudak L., Rouhi G. (2015). The effects of bone screw configurations on the interfragmentary movement in a long bone fixed by a limited contact locking compression plate. *Journal of Biomedical Science and Engineering*.

[13] Heyland M., Duda G. N., Haas N. P. (2015). Semi-rigid screws provide an auxiliary option to plate working length to control interfragmentary movement in locking plate fixation at the distal femur. *Injury*.

[14] Kim J.-D., Kim N.-S., Hong C.-S., Oh C.-Y. (2011). Design optimization of a xenogeneic bone plate and screws using the Taguchi and finite element methods. *International Journal of Precision Engineering and Manufacturing*.

[15] Okumura N., Stegaroiu R., Kitamura E., Kurokawa K., Nomura S. (2010). Influence of maxillary cortical bone thickness, implant design and implant diameter on stress around implants: a three-dimensional finite element analysis. *Journal of Prosthodontic Research*.

[16] Chen S.-H., Chiang M.-C., Hung C.-H., Lin S.-C., Chang H.-W. (2014). Finite element comparison of retrograde intramedullary nailing and locking plate fixation with/without an intramedullary allograft for distal femur fracture following total knee arthroplasty. *The Knee*.

[17] Mehboob H., Chang S.-H. (2015). Optimal design of a functionally graded biodegradable composite bone plate by using the Taguchi method and finite element analysis. *Composite Structures*.

[18] Le T. T., Vo H. V., Webb L. X. (2016). Investigation of Kryptonite™ bone cement in hybrid screw configurations of locking plate humeral midshaft fixation: a study of surrogate bone model. *Journal of Orthopaedics*.

[19] Zhou Y. Z. (2009). A matrix analysis of orthogonal design. *Journal of Mathematics in Practice and Theory*.

[20] El’Sheikh H. F., MacDonald B. J., Hashmi M. S. J. (2003). Finite element simulation of the hip joint during stumbling: a comparison between static and dynamic loading. *Journal of Material Process Technology*.

[21] Alkhodary M. A., Abdelraheim A. E. E., Elsantawy A. E. H. (2015). The development of a composite bone model for training on placement of dental implants. *International Journal of Health Science*.

[22] Sitthiseripratip K., Van Oosterwyck H., Vander Sloten J. (2003). Finite element study of trochanteric gamma nail for trochanteric fracture. *Medical Engineering & Physics*.

[23] He Q., Jiang W., Luo J. (2014). Investigation on biomechanics behavior using three-dimensional finite element analysis for femur shaft fracture treated with locking compression plate. *Journal of Biomedical Engineering*.

[24] Grunwald C., Kaufmann M., Alter B., Vallée T., Tannert T. (2018). Numerical investigations and capacity prediction of G-FRP rods glued into timber. *Composite Structure*.

[25] Chun-hong T., Bo-chu W., Qi C., Li Z., Shao-xi C. (2004). A new experimental design for screening Chinese medicine formula. *Colloids and Surfaces B: Biointerfaces*.

[26] Wei X. L., Li X. R., Wang J. H., Chen Z. W. (2012). Application of matrix analysis in multiple index orthogonal test design. *Advanced Materials Research*.

[27] Cheal E. J., Hayes W. C., White A. A., Perren S. M. (1984). Three-dimensional finite element analysis of a simplified compression plate fixation system. *Journal of Biomechanical Engineering*.

[28] Smith W. R., Stahel P. F., Ziran B. H., Anglen J. O. (2007). Locking plates: tips and tricks. *Journal of Bone & Joint Surgery-American Volume*.

[29] Greiwe R. M., Archdeacon M. T. (2010). Locking plate technology–current concepts. *The Journal of Knee Surgery*.

[30] Sommer C., Gautier E., Müller M., Helfet D. L., Wagner M. (2003). First clinical results of the locking compression plate (LCP). *Injury*.

[31] Chatzistergos P. E., Magnissalis E. A., Kourkoulis S. K. (2010). A parametric study of cylindrical pedicle screw design implications on the pullout performance using an experimentally validated finite-element model. *Medical Engineering & Physics*.

[32] Kohn D., Rose C. (1994). Primary stability of interference screw fixation. *The American Journal of Sports Medicine*.

[33] Mehboob A., Mehboob H., Chang S.-H., Tarlochan F. (2017). Effect of composite intramedullary nails (IM) on healing of long bone fractures by means of reamed and unreamed methods. *Composite Structures*.

[34] Son D.-S., Mehboob H., Chang S.-H. (2014). Simulation of the bone healing process of fractured long bones applied with a composite bone plate with consideration of the blood vessel growth. *Composites Part B: Engineering*.

[35] Mehboob A., Mehboob H., Kim J., Chang S.-H., Tarlochan F. (2017). Influence of initial biomechanical environment provided by fibrous composite intramedullary nails on bone fracture healing. *Composite Structures*.

[36] Son D.-S., Mehboob H., Jung H.-J., Chang S.-H. (2014). The finite element analysis for endochondral ossification process of a fractured tibia applied with a composite IM-rod based on a mechano-regulation theory using a deviatoric strain. *Composites Part B: Engineering*.

